# Deep learning improves the ability of sgRNA off-target propensity prediction

**DOI:** 10.1186/s12859-020-3395-z

**Published:** 2020-02-10

**Authors:** Qiaoyue Liu, Xiang Cheng, Gan Liu, Bohao Li, Xiuqin Liu

**Affiliations:** Department of information and computing science, University of Science and Technology Beijing, Beijing, 100083 China

**Keywords:** sgRNA, Off-target, Deep learning, GloVe model

## Abstract

**Background:**

CRISPR/Cas9 system, as the third-generation genome editing technology, has been widely applied in target gene repair and gene expression regulation. Selection of appropriate sgRNA can improve the on-target knockout efficacy of CRISPR/Cas9 system with high sensitivity and specificity. However, when CRISPR/Cas9 system is operating, unexpected cleavage may occur at some sites, known as off-target. Presently, a number of prediction methods have been developed to predict the off-target propensity of sgRNA at specific DNA fragments. Most of them use artificial feature extraction operations and machine learning techniques to obtain off-target scores. With the rapid expansion of off-target data and the rapid development of deep learning theory, the existing prediction methods can no longer satisfy the prediction accuracy at the clinical level.

**Results:**

Here, we propose a prediction method named CnnCrispr to predict the off-target propensity of sgRNA at specific DNA fragments. CnnCrispr automatically trains the sequence features of sgRNA-DNA pairs with GloVe model, and embeds the trained word vector matrix into the deep learning model including biLSTM and CNN with five hidden layers. We conducted performance verification on the data set provided by DeepCrispr, and found that the auROC and auPRC in the “leave-one-sgRNA-out” cross validation could reach 0.957 and 0.429 respectively (the Pearson value and spearman value could reach 0.495 and 0.151 respectively under the same settings).

**Conclusion:**

Our results show that CnnCrispr has better classification and regression performance than the existing states-of-art models. The code for CnnCrispr can be freely downloaded from https://github.com/LQYoLH/CnnCrispr.

## Background

CRISPR/Cas9 system [1–4] (Clustered regularly interspaced short palindromic repeats /CRISPR-associated 9 system) originally derived from the immune defense mechanism of archaea, it is one of the most popular gene editing technology in recent days. Compared with zinc-finger nucleases [5, 6](ZFNs) and transcription activator-like effector nuclease [6–8](TALENs) technologies, CRISPR/Cas9 system has a pellucid mechanism, simple operation and high efficiency, thus gradually replacing the earlier methods and presently being applied to the fields of biology and clinical medicine, etc.

CRISPR/Cas9 system requires three important components in the process of gene editing: Cas9 protein, guide RNA and PAM motif (protospacer adjacent motif) [9]. Among them, the guide RNA that recognizes a target DNA sequence through complementary base pairing is generally referred to as an sgRNA [10, 11] (single guide RNA, generally an RNA sequence of 20 nt in length). The PAM [11–13] is a 3 nt motif on the target sequence and a prerequisite for Cas9 protein cleavage at a specified site. A common type of PAM is NGG [14–16] (N represents any base of A, T, C, G). During the editing process, the Cas9 protein cleaves the target DNA at the site three bases upstream of the PAM under the guidance of the sgRNA sequence, and performs subsequent gene editing operations: Introduction of an insertion/deletion (indel) base to cause mutation of a gene at a target position by nonhomologous end-joining (NHEJ); or utilization of the “donor template” provided by foreign DNA to recombine with a mutant target to achieve DNA-based editing of the genome by homology-directed repair (HDR) [17–19].

Some studies have found that when CRISPR/Cas9 system operates, several mismatch sites may appear in the complementary matching of sgRNA to the target DNA sequence, therefore resulting in unintended cleavage of the DNA sequence, which is called “off-target” [16, 20, 21]. Fu et al. [20] have confirmed that sgRNA allows 1–5 base mismatches during the guiding process, which in turn causes unintended sequences to be erroneously edited. The existence of off-target phenomenon has greatly hindered the clinical application and further promotion of CRISPR technology. How to assess the off-target propensity of specific sgRNAs and minimize the risk of off-target has become the focus of the CRISPR/Cas9 system study.

Presently, a variety of off-target detection methods have been developed, such as the GUIDE-Seq [22–24] method created by Tsai et al., which can effectively identify 0.1% of mutations in cells and predict the cleavage activity of the system based on sequencing results. The HTGTS [25] method utilizes fusion of known DNA double-strand breaks with other cleavage DNAs to detect DNA breaks by PCR amplification techniques and further detect off-target sites. On this basis, Frock et al. [26] further developed a higher throughput off-target detection method. The BLESS [27] technique further speculate on off-target sites by detecting DNA double-strand breaks. However, this method is complicated to operate and it is impossible to detect a break site that has not occurred or has already been repaired. In addition, the IDLV [28, 29] method can detect off-target sites within the genome-wide range without bias, but with an accuracy of only 1%.

The above detection method cannot detect all off-target sites of a specific sgRNA, and has disadvantages such as high cost, difficult operation, and low detection accuracy. As the core of artificial intelligence, machine learning and deep learning can effectively analyze empirical data and provide important technical support for bioinformatics. To this date, machine learning has been gradually applied to off-target site prediction [14, 30], sgRNA activity prediction [14] and sgRNA design optimization [31, 32], etc. Various machine learning based sgRNA design models [30, 33–36] have been developed and put into application. Their main design idea is to introduce sgRNA sequence features and secondary structure features, rank all possible sgRNA for specific target DNA sequences by scores of off-target effect, and selecting the sgRNA with high cleavage efficiency and low off-target propensity.

The above machine learning methods were based on sequence features. At the time this paper is written, only three existing prediction models have introduced the idea of deep learning into the sgRNA off-target propensity prediction problem.

DeepCpf1 [31], based on the convolutional neural network (CNN), introduced sgRNA sequence features and chromatin accessibility to predict the editing efficiency of sgRNA corresponding to Cpf1. This method does not have to construct the feature artificially, further simplifying the model, and is convenient for researchers to use. DeepCrispr [37] introduced four epigenetic features in addition to DNA sequence features and automatically extracts valid information using the principle of Auto-encoder. Several models including sgRNA target cleavage and off-target propensity prediction were established. However, it is still unknown whether the four epigenetic characteristics will have a positive impact on the model prediction results. CNN_std [38] only used sequence features to construct two-dimensional input matrix by means of “XOR” coding design and utilized CNN for prediction. This deep learning method also received a higher accuracy in the CRISPOR dataset [39]. In addition, Dimauro, G et al. proposed a model named CRISPRLearner [40] for predicting sgRNA on-target knockout activity. Although its purpose is different from ours, its application of deep learning to prediction tasks related to sgRNA provided us with ideas.

Most of the existing prediction methods are still based on machine learning methods and model prediction through complex manual feature extraction [41–46]. However, the internal mechanism of CRISPR gene editing technology is not presently clear and explicit. Manual design of sgRNA features may have a negative impact on the prediction results. Therefore, we would like to present CnnCrispr, a novel computational method for prediction of sgRNA off-target cleavage propensity utilizing the deep learning method. In CnnCrispr, the GloVe embedding model was introduced to extract global and statistical information of input sequences by constructing the co-occurrence matrix of sgRNA and its corresponding DNA sequence. Further integrating with the deep neural network model, the off-target propensity of a given sgRNA at a specific DNA fragments can be predicted. We trained CnnCrispr with the data set used by DeepCrispr [37], and proved that CnnCrispr has a better competitive advantage in predicting sgRNA off-target propensity through performance comparison with four state-of-the-arts models, therefore it is expected to become a potential tool to help on the research of CRISPR system.

## Results

### Model structure and prediction

**Fig. 1:**
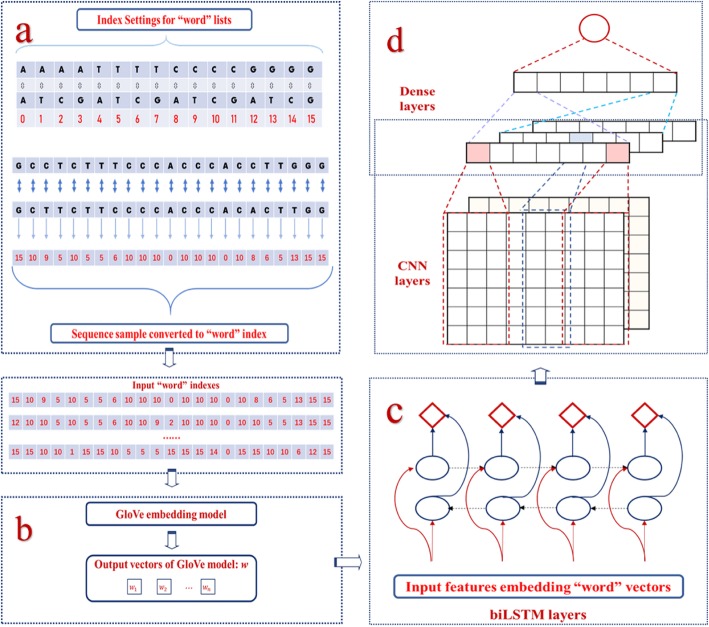
The structure diagram of CnnCrispr. The other four pre-selected models were obtained by adding some parts on the basis of this frame. **a** Rules for setting index values for different *r-d* pair, and an example of index representation for a sgRNA-DNA sequence pair. **b** Based on index representation, the unsupervised GloVe model was used to train the embedded vectors and embed the sequence information into the new input matrix. **c** biLSTM layer was used to capture context information in input information. **d** CNN containing 5 layers with different kernels, scanning the above results to obtain different features. The last fully connected layers were used to obtain the final result

In our initial conception, we combined biLSTM with CNN framework at the final prediction model and the model structure is shown in Fig. 1. We also constructed several similar but different models by removing different network parts to compare the test results and select the final prediction model. All pre-selected network frameworks for model selection are briefly described in Table 1.

**Table 1:** Brief network framework description of several pre-selected models

Model_Name	Description
CnnCrispr	The final model including all units we mentioned
CnnCrispr_NoLSTM	Without biLSTM layer
CnnCrispr_Conv_LSTM	Reverse the order of the convolution layer and the recurrent layer
CnnCrispr_NoBatchNor	Without Batch Normalization layer
CnnCrispr_NoDropout	Without Dropout layer

CnnCrispr is the benchmark model. On this basis, some parts were removed to obtain the comparison models. With the exception of parts mentioned in the description, the structure of the contrast model was completely consistent with the benchmark model. The CnnCrispr_Conv_LSTM model replaced the sequence of convolutional layers and recurrent layer to compare the influence of network sequence on the prediction results

The structure of the benchmark framework of CnnCrispr is described in detail below:

The first layer of CnnCrispr is an embedding layer, which is used for input of the vector obtained by GloVe model. Since the vector dimension of the GloVe model is set to 100, the input of embedding layer is a two-dimensional matrix with the size of 16 × 100. We called the mittens package in Python to train the GloVe model on the basis of the realization of GloVe co-occurrence matrix.

The second layer is a biLSTM network, which is mainly used to extract the context features of input information. Five convolution layers are subsequently connected to the model, and each layer has a different kernel number and kernel size. Then the full connection layers are introduced behind the last convolution layer, having the sizes of 20 and 2 respectively.

In addition to the framework mentioned above, Batch Normalization and Dropout layers are added between each layers to prevent model overfitting. The parameters of the Dropout layer are set as 0.3. For the output layer, *softmax* and *sigmoid* functions are used as activation functions respectively to obtain the prediction results of classification model and regression model.

In the training process, the initial learning rate was set as 0.01, and we used *Adam* algorithm to optimize the loss function. Furthermore, we set the batch size as 256 in consideration of the requirements of potential information extraction from negative data and avoiding the occurrence of over fitting. Too large of a batch size may increase the risk of multiple occurrence of some positive data in a single batch during training, while too small of a batch size may reduce the training speed of a model and extend the training time.

Our experiment was divided into two parts. First, we compared the performance of different models. Then, the final prediction model was compared with the existing models with better performance to evaluate the practical application ability of our model. Detailed network descriptions can be found in Additional file 2.

### Model selection

Experimental data are from the attachment provided by DeepCrispr article, and the relevant data description is detailed in “Data sources” section. During the process of training, 20% of the data in the Hek293t and K562 data sets were randomly selected to compile the test sets (Hek293t test set, K562 test set and Total test set respectively). Different prediction models were obtained by training with all the remaining data, and the prediction performance of each model in the three test sets were evaluated. During the training process, we generated the batch training data using the data sampling method mentioned in “Sampling for training data” section.

We built two models for classification and regression prediction, respectively. The first three models mentioned in Table 1 were trained in order to verify the influence of different parts on the prediction performance of the model. The structure of the benchmark model CnnCrispr is introduced in “Model structure and prediction” section. And the model CnnCrispr_No_LSTM was obtained by removing the LSTM part from the basis of CnnCrispr, CnnCrispr_Conv_LSTM was obtained by adjusting the order of Convolution layers and Recurrent layer on the basis of CnnCrispr. Among them, the purpose of the latter two models was mainly to illustrate whether CNN layer and RNN layer have improved the performance, as well as whether the order of the two frameworks will affect the performance.

We initially trained the three models mentioned above and obtained the prediction results. The model performance is shown in Table 2.

**Table 2:** The prediction results in Model Selection

Test Set	Model	Recall	ROC_AUC	PRC_AUC
Total test set	CnnCrispr	**0.857**	0.975	**0.679**
	CnnCrispr_NoLSTM	0.611	**0.987**	0.651
	CnnCrispr_Conv_LSTM	0.643	0.986	0.67
	CnnCrispr_NoBatchNor	–	0.5	0.504
	CnnCrispr_NoDropout	0.810	0.985	0.625
Hek293t test set	CnnCrispr	**0.864**	0.971	0.686
	CnnCrispr_NoLSTM	0.631	**0.988**	0.658
	CnnCrispr_Conv_LSTM	0.660	0.988	**0.694**
	CnnCrispr_NoBatchNor	–	0.5	0.504
	CnnCrispr_NoDropout	0.816	0.988	0.636
K562 test set	CnnCrispr	**0.826**	**0.995**	**0.688**
	CnnCrispr_NoLSTM	0.522	0.985	0.589
	CnnCrispr_Conv_LSTM	0.565	0.981	0.57
	CnnCrispr_NoBatchNor	–	0.5	0.503
	CnnCrispr_NoDropout	0.783	0.973	0.597

CnnCrispr had the best comprehensive performance in the three test sets, and the calculated recall value was higher than other pre-selected models. The numbers in boldface indicate the highest scores for each metric

**Fig. 2:**
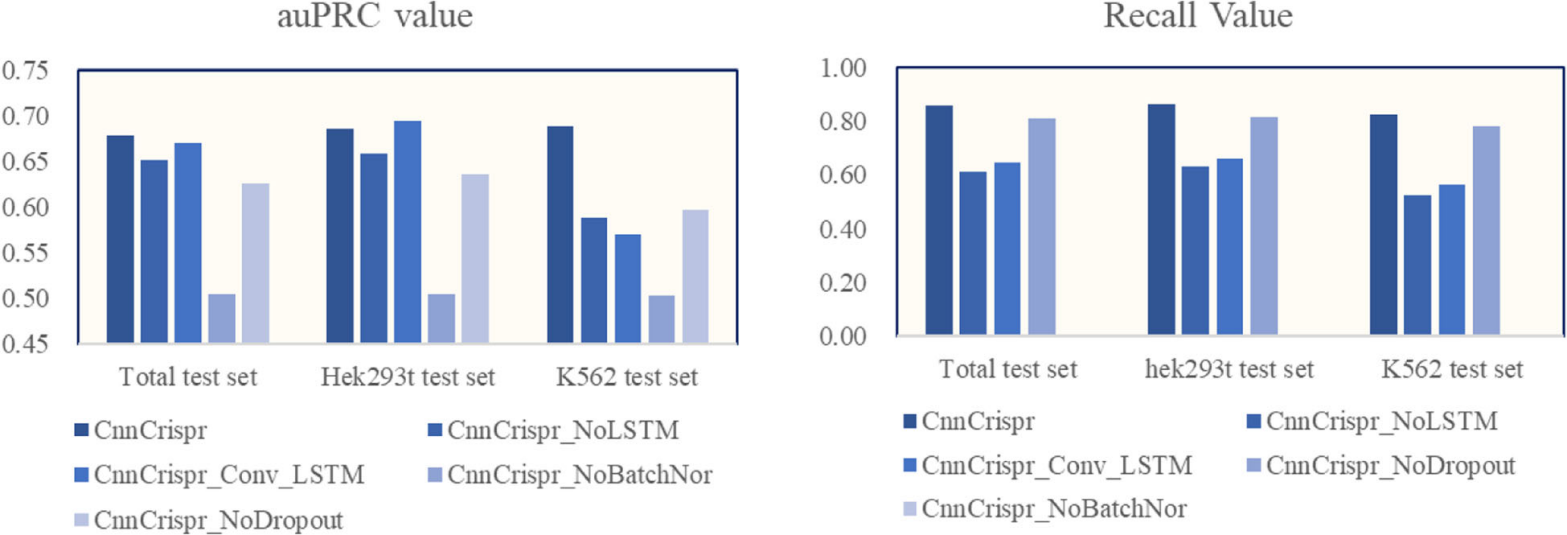
Visualization of auPRC and Recall value in three test sets. CnnCrispr had higher auPRC and Recall values in the three test sets. Although the performance of CnnCrispr_NoDropout was similar to CnnCrispr, the addition of Dropout layer can reduce the network parameters, shorten the training duration and prevent over-fitting of the model to some extent, hence we finally chose the CnnCrispr with Dropout layer

Due to the highly unbalanced nature of the data set, it was easy for the model to obtain a high auROC value. Therefore, we gave up the comparison of auROC values and focused on the comparison results of auPRC and Recall value on the test set. The results in Table 2 were used to draw the histogram (Fig. 2), from which it can intuitively be seen that CnnCrispr has better predictive performance. Therefore, we took the CnnCrispr as the benchmark network framework and further well-tuned the network structure.

Based on CnnCrispr, the Dropout layer and Batch Normalization layer were removed respectively to verify the influence of the two parts on performance. A brief description of the network structure is given in Table 1. The recall value of CnnCrispr_No_Dropout was 0.810 in the total test set, which was a little lower than that of CnnCrispr, this showed that the Dropout layer does have improved performance and prevented over-fitting, although the degree of improvement is not very noticeable. However, after adding the Dropout layer, the training parameters of the model were greatly reduced, which further saved time for model training, hence we kept the Dropout layer in the final model. Then we trained the model without the Batch Normalization layer several times and analyzed it on the test set, but every time the entire test set were all classified as negative samples. This indicated that the model without the BN layer has lost its ability of classification prediction. Therefore, the BN layer is essential in the final model. In addition, we also mentioned the importance of BN layer for neural network model in “Convolution neural network and batch normalization” section, hence we reserved it in our final model.

### Model comparing

We selected four sgRNA off-target propensity prediction models for model comparison, namely CFD [33], MIT [16], CNN_std [38] and DeepCrispr [37].

CFD is short for Cutting Frequency Determination. As a scoring model for evaluating the off-target propensity of sgRNA-DNA interaction, CFD specified different scores for the location and type of mismatch between sgRNA and corresponding DNA sequence. When multiple mismatches appear in the sequence pair, the corresponding scores are multiplied to obtain the final score. For example, if the sgRNA-DNA sequence has a rG-dA mismatch in position 6 and a rC-dT mismatch in position 10, it will receive a CFD score of 0.67 × 0.87 = 0.583. Haeussler et al. [39] compared the performance of CFD with that of MIT, and proved that the prediction performance of CFD was slightly better than that of MIT in CRISPOR data set. CNN_std is a CNN-based sgRNA off-target propensity prediction model developed by *Jiecong Lin*. The combination of sgRNA and corresponding DNA sequences was encoded by “XOR” principle and predicted by multi-layer convolution network. DeepCrispr is a deep learning method which combines sgRNA-DNA sequence information with genomic epigenetic characteristics as the input. DeepCrispr used the largest data set available to conduct model training and introduced the auto-encoder to automatically acquire potential features of the sgRNA-DNA sequence, which was a good attempt at deep learning in sgRNA related prediction problems.

In order to make a more comprehensive comparison with the four models above, we tested the performance of the classification and regression models in two test patterns. We downloaded the prediction models of CFD, MIT and CNN_std from relevant websites and obtained the prediction results on the same test set as CnnCrispr. Due to the fact that the training methods were consistent between CnnCrispr and DeepCrispr, we just used the test results given by DeepCrispr to make the comparison.

#### Test pattern 1 -- withheld 20% as an independent testing set

**Fig. 3:**
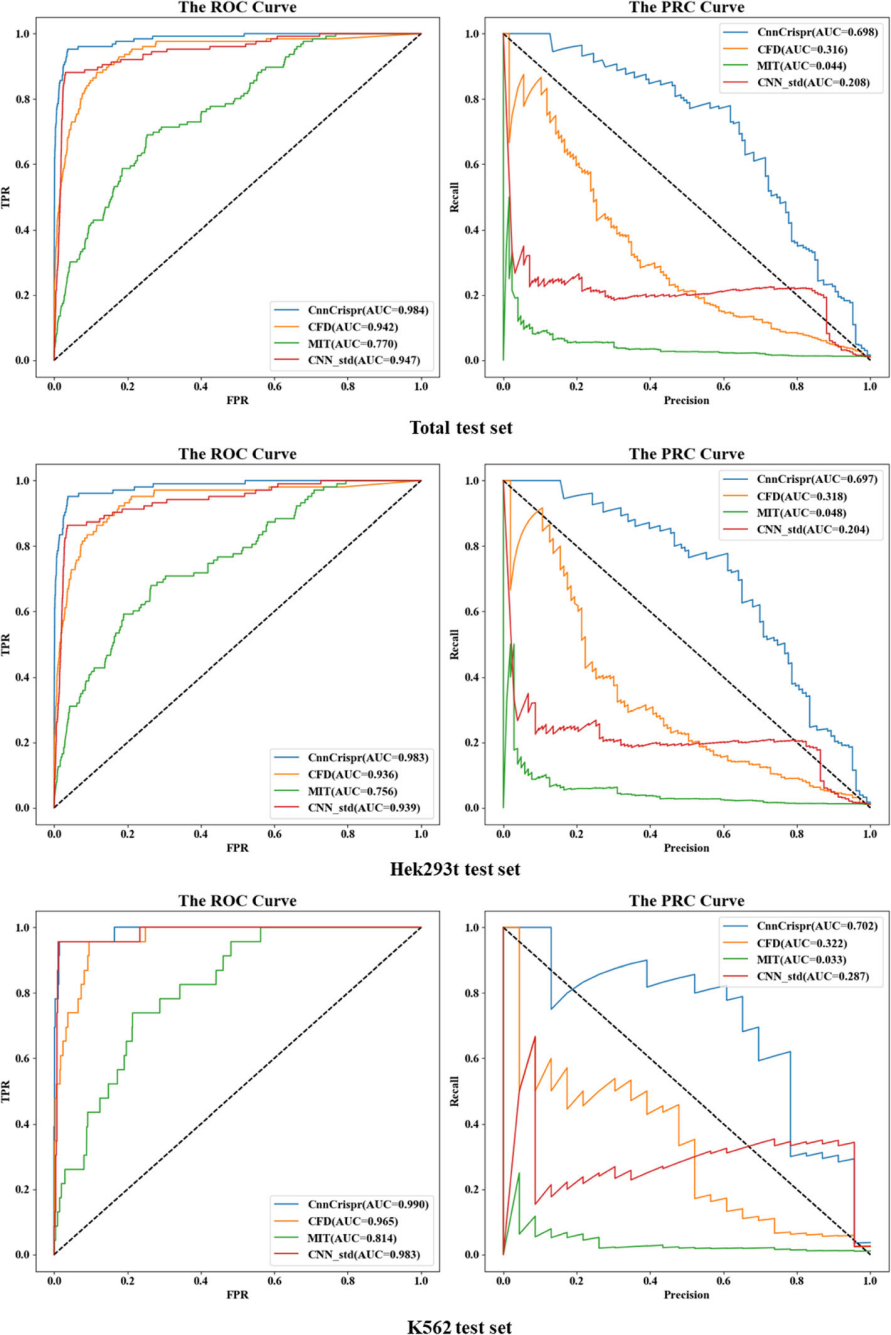
ROC curves and PRC curves of CnnCrispr and three state-of-the-arts prediction models including CFD, MIT and CNN_std under test pattern 1

Consistent with the training method of “Model Selection” section, we randomly divided the data sets of each cell line in the proportion of 8:2. We compared the performance of CnnCrispr with the current preferable prediction models. Fig. 3 shows the comparison results under the classification schema. CnnCrispr achieved an auROC value of 0.975 and an auPRC value of 0.679 at the total test set. Which were both higher than the value of CFD, MIT and CNN_std (there were similar trends in the Hek293t test set and K562 test set, CnnCrispr achieved the auROC of 0.971 and 0.995 on Hek293t test set and K562 test set, respectively. And auPRC of 0.686 and 0.688 on Hek293t test set and K562 test set, respectively). The AUC values of ROC curve and PRC curve of CnnCrispr on the three test sets were all higher than those of CFD, MIT and CNN_std, which proved that CnnCrispr had more advanced prediction ability. In addition, the PRC curve obtained by CnnCrispr on the total test set and Hek293t test set completely contained the PRC curve obtained by the other three models, CFD, MIT and CNN_std, while on the K562 test set, only a small portion of the curve was covered by the CNN_std. Comprehensive comparison showed that the overall performance of the CnnCrispr was better than the other three models, and since the training and test sets were extremely unbalanced, the PRC curve and the area under it were more important measures for model evaluation, where CnnCrispr had a strong competitive advantage. In addition to the comparison with the above three models, we further compared the testing performance of CnnCrispr with DeepCrispr. Since the training methods and data sets were consistent, we directly compared the test results given in ref. [37], and the results are shown in Table 3. The auROC values of DeepCrispr were slightly better than those of CnnCrispr (shown more intuitively on Hek293t test set), but the auPRC values obtained by CnnCrispr on all three test sets were higher than those of DeepCrispr. By comprehensive comparison, CnnCrispr showed better performance than DeepCrispr under test pattern 1.

**Fig. 4:**
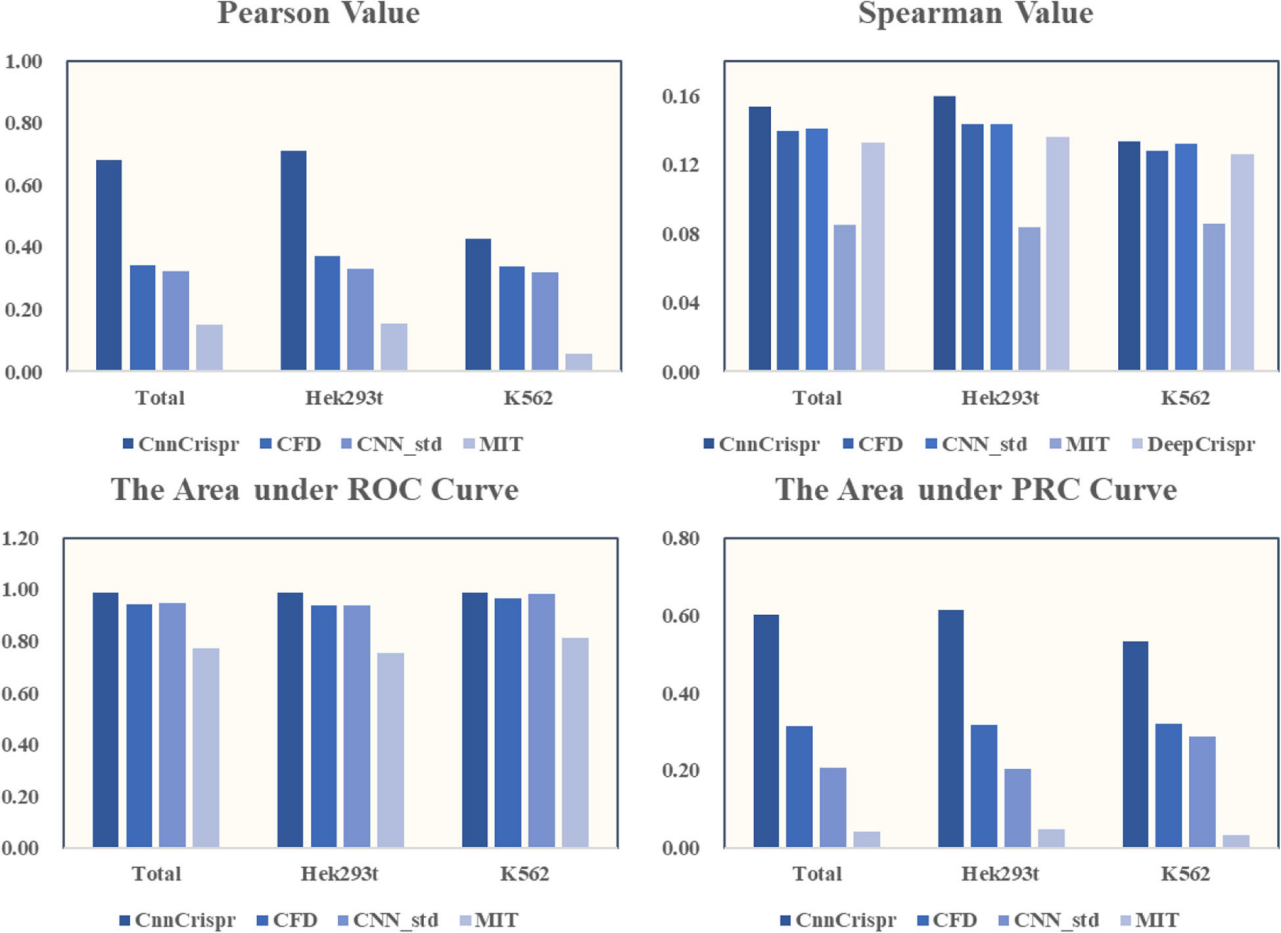
Performance comparison results of CnnCrispr and states-of-the-arts prediction models under test pattern 1. The two figures above show the Pearson and Spearman values obtained by regression schema, the two figures below show the AUC values under ROC and PRC curves by referring to the CRISTA’s evaluation method

**Table 3:** Performance comparison with states-of-the-art models under test pattern 1

Test Set	Model	auROC	auPRC	Pearson value	Spearman value
Total test set	CnnCrispr	0.975	**0.679**	**0.682**	**0.154**
	CFD	0.942	0.316	0.343	0.140
	MIT	0.77	0.044	0.150	0.085
	CNN_std	0.947	0.208	0.321	0.141
	DeepCrispr	**0.981**	0.497	–	0.133
Hek293t test set	CnnCrispr	0.971	**0.686**	**0.712**	**0.160**
	CFD	0.936	0.318	0.371	0.143
	MIT	0.756	0.048	0.153	0.084
	CNN_std	0.939	0.204	0.330	0.144
	DeepCrispr	**0.984**	0.521	–	0.136
K562 test set	CnnCrispr	**0.995**	**0.688**	**0.426**	**0.134**
	CFD	0.965	0.322	0.336	0.128
	MIT	0.814	0.033	0.057	0.086
	CNN_std	0.983	0.287	0.319	0.132
	DeepCrispr	0.953	0.41	–	0.126

We downloaded the prediction models of CFD, MIT and CNN_std from relevant websites and obtained the prediction results on the same test set as CnnCrispr. Since the training process of CnnCrispr was consistent with DeepCrispr’s, we directly used the test results in Additional file 2 given by DeepCrispr for performance comparison. The numbers in boldface indicate the highest scores for each metric

Unlike the classification schema, the Pearson correlation coefficient and Spearman rank correlation coefficient of the prediction results were mainly used as evaluation measures for regression schema. From the comparison results, the Pearson correlation coefficient between CnnCrispr’s prediction results and the real labels was strictly superior to the three comparison models (Since the Pearson coefficient was not selected as the evaluation measure in DeepCrispr, we only compared the Spearman values of CnnCrispr with DeepCrispr.).

In Fig. 4, The Pearson value of CnnCrispr on Hek293t test set reached 0.712(higher than 0.371 obtained by CFD, 0.153 obtained by MIT, 0.33 obtained by CNN_std). In the entire test set, CnnCrispr also demonstrated its better predictive ability, with Pearson value reaching 0.682, higher than 0.343 of CFD, 0.150 of MIT and 0.321 of CNN_std. For Spearman correlation coefficient, the negative data in the test set was much larger than the positive data (about 250:1), therefore, a high Spearman value cannot be achieved. Nevertheless, the prediction ability of CnnCrispr was still better than those of the four models above (the test results of CnnCrispr on Hek293t, K562 and Total test set were 0.154, 0.160 and 0.134 respectively, while the Spearman correlation coefficients of CFD on the three test sets were 0.140, 0.143 and 0.128 respectively; Spearman correlation coefficients of MIT were 0.085, 0.084 and 0.086 respectively; Spearman correlation coefficients of CNN_std were 0.141, 0.144 and 0.132 respectively; Spearman correlation coefficients of DeepCrispr were 0.136, 0.126 and 0.133 respectively). In addition, we also compared the AUC values under ROC and PRC curves of the five models by referencing the CRISTA’s evaluation method and considering the predicted results as the probability of the classification labels. The auROC value and auPRC value obtained by CnnCrispr on the total test set were as high as 0.986 and 0.601 respectively, which were superior to 0.942 and 0.316 of CFD, 0.947 and 0.208 of CNN_std, and the same results were obtained on Hek293t and K562 test sets. Based on the above performance results, we concluded that CnnCrispr had better prediction ability.

#### Test pattern 2 – “leave-one-sgRNA-out”

In order to examine the accuracy and generalization ability of CnnCrispr for the prediction of off-target propensity of new sgRNA, we set up the “leave-one-sgRNA-out” experiment, which is a good evaluation method for the prediction of off-target propensity. During the training, a sgRNAs and its corresponding off-target sequences (with true cleaved propensity or the potential sites obtained from whole genome) were completely extracted for model testing. According to the difference of sgRNAs, model training and performance evaluations were conducted a total of 29 times. Through this 29-fold cross-validation method, we were able to comprehensively evaluate the generalization ability of CnnCrispr and avoid over-fitting or under-fitting of the model when predicting for some special sgRNAs.

**Fig. 5:**
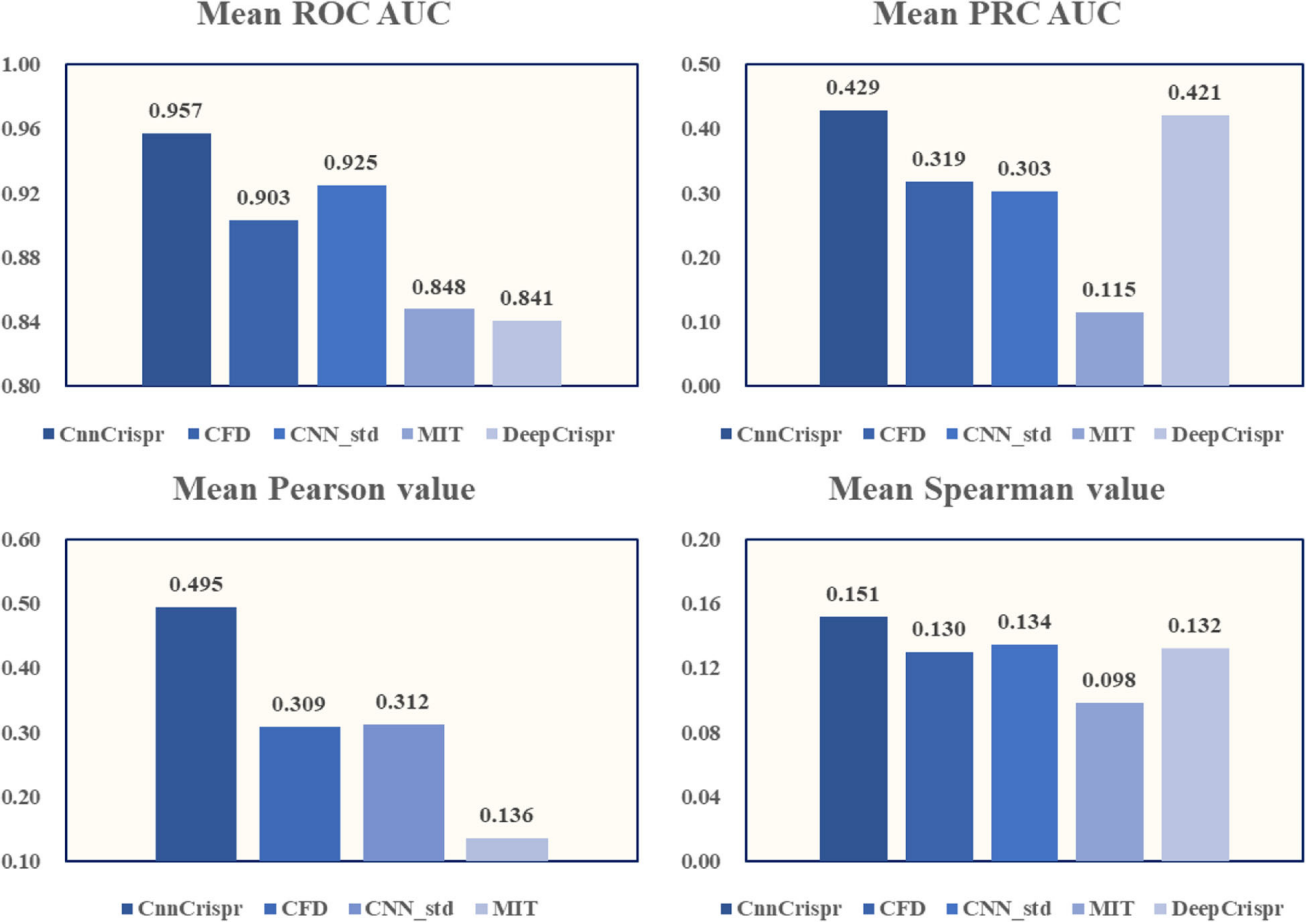
Leave-one-sgRNA-out comparison of sgRNA off-target propensity prediction with auROC, auPRC, Pearson correlation coefficient, and Spearman correlation coefficient. The bar chart shows the mean value obtained by 29-fold cross validation. The two figures above show the performance comparison results in classification schema and the two figures below shows the performance comparison results in regression schema

For classification, CnnCrispr achieved an average auROC of 0.957 and auPRC of 0.429, which were both higher than the results of the four models above (CFD achieved an average auROC of 0.903, auPRC of 0.319, MIT achieved an average auROC of 0.848, auPRC of 0.115, CNN_std achieved an average auROC of 0.925, auPRC of 0.303; and DeepCrispr achieved an average auROC of 0.841, auPRC of 0.421). In the 29-fold cross validation, CnnCrispr’s comprehensive competitive advantage was more significant, and the auPRC results were higher than results yielded by the other four models, which was essential to prevent the model from missing the actual off-target sites (see Fig. 5).

**Fig. 6:**
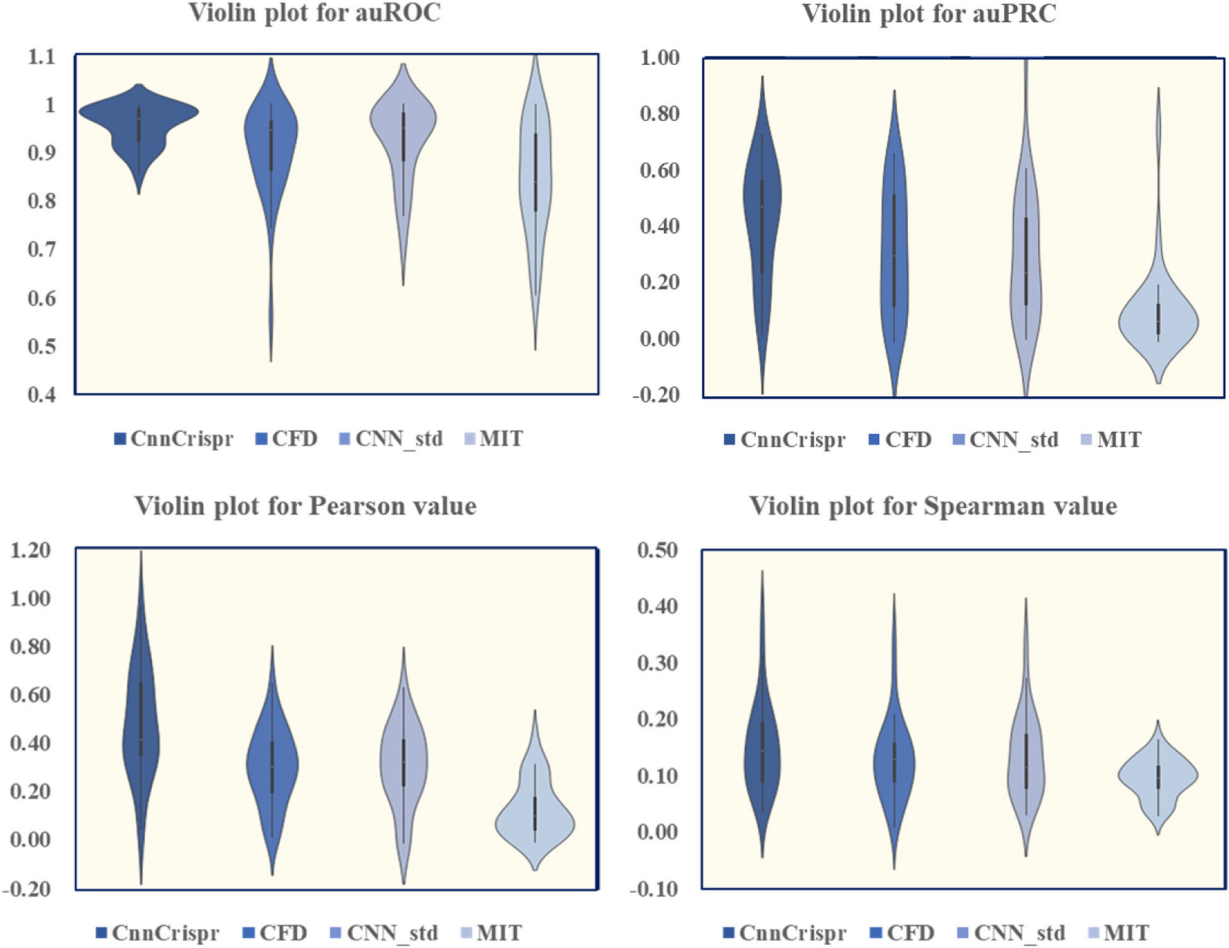
Leave-one-sgRNA-out comparison of sgRNA off-target efficacy prediction with auROC, auPRC, Pearson correlation coefficient, and Spearman correlation coefficient. The 29 test results were plotted as the violin plots. The two figures above show the performance comparison results in classification schema and the two figures below shows the performance comparison results in regression schema. It is worth noting that the article of DeepCrispr did not offer the detailed test results of leave-one-sgRNA-out validation, as a result we did not draw the corresponding violin plot. It is anticipated that this comparison process can further improve upon future works

In order to make a more comprehensive evaluation, we also considered the distribution of the values of auROC and auPRC obtained by “29-fold” cross-validation, and drew the violin plot (Due to the fact that we weren’t able to get the test data of DeepCrispr, we were unable to draw a violin plot for it.). Violin plot is characterized by the kernel density estimation of the basic distribution, and the external shape of the violin plot is the kernel density estimation. First of all, Fig. 6 shows that the auROC values of CnnCrispr were generally higher and the AUC values of CnnCrispr were more concentrated, 75% of the prediction results were greater than 0.9. On the other hand, there were obvious abnormal points in the prediction results of auROC by the other three models, indicating that they cannot play a good role in predicting the off-target propensity of individual sgRNA. In addition, the distribution of CnnCrispr’s auROC values was more concentrated, while the auROC values of CFD and CNN_std had obvious discrete values (the whiskers on the lower side were longer). With the increase of auROC values, the horizontal distance of the violin plot plotted by CnnCrispr was larger, which showed that more auROC values were distributed on this interval, further indicating the good prediction performance of CnnCrispr. For auPRC values, the median of prediction results obtained by CnnCrispr was significantly larger than that of the other three models, which showed that CnnCrispr had a higher overall score and 75% of auPRC values obtained by CnnCrispr were greater than 0.2. CnnCrispr was more distributed at higher scores, indicating that the overall predictive performance of CnnCrispr was indeed better than that of CFD and CNN_std (see Fig. 6).

We further compared the 29-fold cross-validation results in regression schema and organized the performance visualization results in Fig. 5-6. We first compared the average value of Pearson correlation coefficient and the Spearman correlation coefficient (see Fig. 5). CnnCrispr achieved a higher mean Pearson value and Spearman value, this showed that CnnCrispr had better fitting ability. Furthermore, we drew 29 sets of Pearson values and Spearman values into violin maps. As shown in Fig. 6, Pearson values obtained by CnnCrispr were more distributed in the high score range. In addition, the Spearman scores of all four models were lower, but despite this, the distribution of CnnCrispr scores was significantly better than that of the other three models. Concluding with the fact CnnCrispr had a higher probability of obtaining highly fitting prediction results for off-target propensity (Detailed results are in Additional file 1).

## Discussion

As a kind of classical neural network algorithm, RNN has the following features: memory ability, Shared parameters and Turing completeness. Therefore, it has advantages in learning the nonlinear features of sequences and plays an important role in the study of sequence problems with time characteristics. In the relevant studies of CRISPR editing technology, it has been shown that the base types at different positions have a certain influence on the cleavage propensity of sgRNA [11, 21, 41, 42, 47]. Therefore, we considered introducing an RNN framework into the prediction model to extract context information for sgRNA-DNA pairs.

The convolution kernel size of the CNN was smaller than the input matrix, so the convolution operation can extract more local features -- which is consistent with the image processing. In fact, it is not necessary for each neuron to perceive the global image, but only need to perceive the local image, and then integrate the local information at a higher level to obtain the global information. The parameter sharing mode of CNN can also greatly reduce the computation. In addition, we set convolution kernels of different sizes for different levels in the convolution part, and used multiple convolution kernels to convolve the input images, to extract local features as comprehensively as possible in this way. Furthermore, GloVe method utilized the statistical information of global word co-occurrence to learn word vectors, so as to combine the advantages of statistical information with the local context window method. We used this method to replace the traditional “one-hot” representation method hence allowing the input sequence of CnnCrispr to have better characteristic representation ability.

In the initial structural design of the model, we comprehensively considered the necessity of extracting sequence context information and local region information, so we integrated RNN and CNN model to improve the ability of feature extraction, and the excellent prediction ability of the final network model CnnCrispr was proved by comparing with the performance of different pre-selected models. The final network structure is shown in Fig. 1. After the GloVe model, the biLSTM was connected to extract context features, and the two-dimensional matrix information was further extracted by using 5 convolutional layers. In the output layer of the network, the model was divided into classification schema and regression schema by setting different activation functions (*softmax* or *sigmoid* functions).

In “Model Selection” section, we also intuitively saw that the order of RNN and CNN had a great impact on the test performance, and the model CnnCrispr_Conv_LSTM cannot play a very good role in feature extraction and data prediction (see **“Model selection” section** and Table 2). We briefly analyzed the following reasons: the RNN can fully extract the contextual text features of input sequences, while the convolution operation will initially break the internal connection of sequences and affect the function of RNN. Firstly, the RNN operation was carried out to extract the context features of the sequence, and then the CNN was used to extract the local features, and the local information was integrated at a higher level to obtain the global feature information, so as to improve the prediction ability of CnnCrispr.

In comparison with the performance of the existing four state-of-the-arts prediction models, CnnCrispr had better prediction ability in highly unbalanced test sets from DeepCrispr. In the “leave-one-sgRNA-out” experiment, the mean auPRC of 0.471 and mean Pearson value of 0.502 were achieved, which showed that CnnCrispr has a better competitive advantage. In addition, CnnCrispr only used the sequence information between sgRNA and corresponding potential DNA segments, giving up the construction of artificial features, thus avoiding the introduction of invalid or interfering information and making the prediction results more convincing.

We hope that CnnCrispr can help clinical researchers narrow down the screening range of off-target site test and save researchers more time and energy.

Since 2014, the number of open source data sets and online resources available for studying of the application of machine learning on CRISPR/Cas9 system has been increasing. As of the day this composition is written, the data set used by the author for model training is the largest data set presently available. However, with the continuous development of biological research technology, the number of available open source data sets will gradually increase, this will further improve the generalization ability of CnnCrispr in the future.

## Conclusion

In this paper, we built a novel sgRNA off-target propensity prediction model, CnnCrispr. With introduction of the GloVe model, CnnCrispr attempted new feature representation methods to embed sequence information into the deep learning model, combined RNN with CNN, and only used sequence information to predict the off-target propensity of sgRNA at specific sites. By comparison with existing prediction models, the superior prediction ability of CnnCrispr was further confirmed. Our model used deep learning to comprehend the automatic learning of sequence features between sgRNA and corresponding potential off-target site, avoiding the unknown influence of artificial feature construction process on model prediction results, which is a new attempt at deep learning in the direction of sgRNA off-target propensity prediction.

## Methods

### Data sources

So far, there is no public website to integrate off-target data, and most studies still use various detection methods to obtain the data of potential off-target sites and off-target propensity of specific sgRNA at specific site. We used the off-target data that has been published in the DeepCrispr article as our training data. The off-target data set contains a total of 29 sgRNA from two different cell types: 293-related cell lines and K562 t. For all 29 sgRNAs, a total of more than 650 positive data have been identified as off-target sites, and Guohui C et al. [37] obtained more than 160,000 possible loci across the whole genome similar to the corresponding sgRNA using *bowtie2*. The whole dataset was highly unbalanced, it was likely to affect model fitting precision in the process of training, we will further describe the concrete solution in the “Sampling for Training data” section. For the classification model, the labels of off-target sites were set to “1”, and the labels of other sites were set to “0”.For the regression model, the labels of off-target site were set to the targeting cleavage frequency detected by different detection assays and other sites were set to “0”.

### Data preprocessing and encoding

The sgRNA sequence and its corresponding DNA sequence are each composed of 23 bases. Considering that the combination of the two bases at the same site is a feature, since the DNA sequence is composed of four types of bases: A, C, G and T, there are altogether 4 × 4 = 16 possible situations for this feature. Therefore, we set a unique index value for each combination, and encoded the original sgRNA-DNA sequence with an index vector of 23 for subsequent GloVe model embedding (showed in Fig. 1a).

### GloVe model for data embedding

As an unsupervised word representation method, the GloVe [48] model enables vectors to contain as many hidden features of input data as possible through vectorization of the original data, which can eliminate the disadvantages of the one-hot coding method. In this model, the input vector was obtained by multiplying the one-hot coding of the original “vocabulary” by a trained weight matrix. When training the GloVe model, the co-occurrence matrix *X* should firstly be calculated according to the original “corpus” (here it refers to the original data set composed of our original sgRNA and corresponding off-target sequences). An element *x*_*i*, *j*_ in matrix *X* is the sum of the times that the word *w*_*j*_ appears in the context box of the word *w*_*i*_.

${X}_i=\sum \limits_{j=1}^N{x}_{i,j}$, represents the sum of the times that appear in the context box of word *w*_*i*_ for all words in the word list.

${P}_{i,k}=\frac{x_{i,k}}{X_i}$, represents the probability that word *w*_*k*_ appears in the context box of word *w*_*i*_.

$rati{o}_{i,j,k}=\frac{P_{i,k}}{P_{j,k}}$, represents the correlation of words *w*_*i*_, *w*_*j*_, *w*_*k*_ (Table 4). We can notice that the value of *ratio*_*i*, *j*, *k*_ is calculated according to the co-occurrence times in the co-occurrence matrix *X* = (*x*_*i*, *j*_). Now we hope to construct a word vector for each word and reproduce the value by using the word vector *v*_*i*_, *v*_*j*_, *v*_*k*_ and a specific function *g*(•). If such a word vector *v*_*i*_ can be found, it indicates that the word vector will definitely contain the information in the co-occurrence matrix.

**Table 4:** Different ratios indicate the possible relationship between three words, *w*_*i*_, *w*_*j*_, *w*_*k*_

*ratio*_*i*, *j*, *k*_	*w*_*i*_ is related to *w*_*k*_	*w*_*i*_ is not related to *w*_*k*_
*w*_*j*_ is related to *w*_*k*_	Tends to 1	Very small and tends to 0
*w*_*j*_ is not related to *w*_*k*_	Greater than 1	Tends to 1

In order to make the value of *g*(*v*_*i*_, *v*_*j*_, *v*_*k*_) as close as possible to $rati{o}_{i,j,k}=\frac{P_{i,k}}{P_{j,k}}$, we considered to build a cost function:

$$ J=\sum \limits_{i,j,k}^N{\left(\frac{P_{i,k}}{P_{j,k}}-g\left({v}_i,{v}_j,{v}_k\right)\right)}^2, $$

and obtained the final cost function expression:

$$ J=\sum \limits_{i,j}^Nf\left({x}_{i,j}\right){\left({v_i}^{\mathrm{T}}{v}_j+{b}_i+{b}_j-\log \left({x}_{i,j}\right)\right)}^2 $$

through a series of hypothesis derivation.

In order to implement the GloVe embedding model, we called the *Python* extension package *mittens*, and used the GloVe model to train the preprocessed co-occurrence matrix. Finally, the embedded word vector representation was obtained. Global Vector integrated the global statistics of Latent Semantic Analysis (LSA) with the advantages of local context window. By integrating the aforementioned statistical information in its entirety, the training speed of the model can be accelerated and the relative weight of words can be controlled.

### Recurrent neural network and LSTM variant

As a special neural network model, the recurrent neural network (RNN) adds the horizontal connection of each neuron node in the same layer on the basis of the multi-layer feedforward network.

$$ {\displaystyle \begin{array}{l}{O}_t=g\left(V{S}_t\right)\\ {}{S}_t=f\left(U{x}_t+W{S}_{t-1}\right)\end{array}} $$

RNN is mainly used to process time series. The output layer performs a full connection operation with the adjacent hidden layer. *V* is the connection matrix of the output layer, and *g* is used as an activation function to obtain the final output result. For the hidden layer at time *t*, neuron node first receives the network input from that moment by weight *U* and receives the hidden layer output from time *t* − 1 by weight *W*. And further operations on the sum of the two are taken. It can be seen that RNN has only one hidden layer, and the hidden layer is called multiple times during the training process to realize the information extraction function of the input information context. RNN implements the memory function but this memory function is limited because there will be a phenomenon of gradient disappearance or gradient explosion. Therefore, *Hochreiter* and *Schmidhuber* [49] proposed the LSTM network to solve the above mentioned problems by introducing a memory cell, the key to which is the cell state, which receives or rejects input information by a well-designed structure called a “gate”. Although many LSTM variants have been proposed, we have only described the forward recursion of a most basic LSTM model. The basic formulas were given below:

Input gate: *f*_*t*_ = *σ*(*W*_*f*_ · [*h*_*t* − 1_, *x*_*t*_] + *b*_*f*_).

Forgetting gate: *i*_*t*_ = *σ*(*W*_*i*_ · [*h*_*t* − 1_, *x*_*t*_] + *b*_*i*_)

$$ {\tilde{C}}_t=\tanh \left({W}_C\cdotp \left[{h}_{t-1},{x}_t\right]+b{}_C\right) $$

Output gate: *o*_*t*_ = *σ*(*W*_*o*_ · [*h*_*t* − 1_, *x*_*t*_] + *b*_*o*_).

For the model input at time *t*, the input gate controls the selection of information and adds it to next step. The value of the input gate is usually between [0, 1] as the degree of information selection. The purpose of forgetting gate is mainly to prevent the introduction of too much information to relieve the burden of memory *C*. Therefore, some useless information is discarded by setting forgetting gate. As a deformed structure of RNN, LSTM adds memory units to each neuron in the hidden layer. Through the setting of several controllable gates on the neuron, it can control the memory and forgetting degree of current information and previous information, thus achieving the function of long-term memory.

In order to get the characteristic representation of forward and backward information of RNA sequences, the bidirectional LSTM (showed in Fig. 1c), a variant of LSTM, was used, which consists of two parallel LSTM: one input forward sequence and one input reverse sequence. Here’s how it works:

$$ {y}_2=g\left(V{A}_2+{V}^{\hbox{'}}{A}_2^{\hbox{'}}\right) $$

And,

$$ {\displaystyle \begin{array}{l}{A}_2=f\left(W{A}_1+U{x}_2\right)\\ {}{A}_2^{\hbox{'}}=f\left({W}^{\hbox{'}}{A}_3^{\hbox{'}}+{U}^{\hbox{'}}{x}_2\right)\end{array}} $$

For output *y*_2_, its input mainly comes from the weighted sum of two parts, *A*_2_ and ${A}_2^{\hbox{'}}$. Therefore, two values should be stored in the hidden layer of LSTM, which are respectively *A* participating in forward calculation and *A*^'^ participating in reverse calculation.

### Convolution neural network and batch normalization

CNN (showed in Fig. 1d) mainly uses image data as network input, which avoids the complex process of feature extraction and data reconstruction that often occurs in traditional recognition algorithms. Therefore, it has great advantages in 2D image processing.

In the training of CnnCrispr, due to the small number of samples available for training and testing, the samples in the training set will be over-trained, which will lead to overfitting problem and the insufficient generalization ability of the model in the test and other related data sets. Therefore, we need to reduce the over-fitting phenomenon of the model as much as possible by adjusting the model structure. Batch Normalization is a good tool for solving this problem. With the deepening of network depth or in the training process, the distribution of the activated input value before the nonlinear transformation will gradually shift or change the deep neural network, and the gradient of the low layer neural network will disappear in the backward propagation process, hence the convergence speed of the deep layer neural network will decrease. Batch Normalization forcibly transforms the input value distribution of any neuron into the standard normal distribution with a mean value equaling to 0 and variance equaling to 1, ensuring that the input value of each layer of network is uniformly distributed in the training process of the model, so as to avoid the gradient disappearance problem and further accelerate the training speed. In addition, the Batch Normalization layer, as an alternative operation to Dropout, avoids the inactivation of some input nodes and thus reduces the loss of effective information.

### Sampling for training data

The samples in the entire data set is extremely unbalanced: the number of negative samples is about 250 times that of positive samples. A highly unbalanced data set may make the gradient update process unstable, increase the difficulty of training, and even lead to the failure of model training. In order to solve this problem, we designed a data sampling method for model training: we set the batch size as *m* for model training, and divided the negative sample set into *N* subsets according to this value (if the negative set has *M* samples, batch size is *m*, then *N* =[*M* / *m*]), and *N* random oversampling operations are further performed on the positive training set, therefore generating *N* training batches. In the data sampling process, almost all negative data are traversed, and the potential information of the negative data can be searched more reliably. However, one thing to note is that oversampling may over-emphasize the effects of some positive data on the training results, resulting in the model overfitting. So, the choice of batch size is very crucial.

### Model evaluation and performance measures

In this paper, we investigated the classification and regression models for prediction of off-target propensity. For the classification model, the confusion matrix *M* of the predicted results can be obtained.

$$ M=\left[\begin{array}{cc} TP& FN\\ {} FP& TN\end{array}\right] $$

Among them, *TP* (True Positive) and *TN* (True Negative) were expected prediction results, while *FP* (False Positive) and *FN* (False Negative) would reduce the prediction accuracy of our model.

For the prediction of off-target propensity, we should pay more attention to whether the actual off-target site is correctly predicted as “off-target” -- that is, whether the label value is 1. Therefore, ROC curve, PRC curve and Recall value were selected as the performance measures. The Recall value is defined as follows:

$$ Recall=\frac{TP}{TP+ FN} $$

The recall value can well describe the proportion of the actual off-target sites that are correctly classified, that is, the larger the recall value is, the better the prediction ability of the model will be.

ROC and PRC curves [50] are widely used in classification model, it is important to note that since the prediction problem of category has highly unbalanced characteristics, compared with the AUC value under the ROC curve, the area under the PRC and its curve is more worthy of attention, the higher the value, proves the model has better performance in class imbalance problems.

For the regression model, the Pearson and Spearman correlation coefficients are mainly considered as metrics. The two measures are continuous variable correlation evaluation indicators, between that two, the Pearson value pay more attention to whether it has a linear relationship between two variables, while Spearman value is a nonparametric statistical method to do the linear correlation analysis by using the rank of each variable.

## Supplementary information

**Additional file 1.** Detailed comparison results for sgRNA off-target propensity prediction.

**Additional file 2.** A detailed description of the model structure for model selection.

## Data Availability

All data used during this study are included in the published article “DeepCrispr” and its supplementary information files (DOI:10.1186/s13059-018-1459-4). We obtained 29 sgRNAs and their corresponding DNA sequences from its Additional file 2. The data can also be downloaded from https://github.com/LQYoLH/CnnCrispr and the file name is “off-target_data”.

## Abbreviations

auPRC: Area under the PRC curve
auROC: Area under the ROC curve
biLSTM: Bi-directional Long-Short Term Memory
CNN: Convolutional neural network
CRISPR/Cas9 system: Clustered regularly interspaced short palindromic repeats /CRISPR-associated 9 system
GloVe: Global vector
PAM: Protospacer adjacent motif
RNN: Recurrent neural network
sgRNA: Single-guide RNA
