# Supplementary material for: Deep learning improves the ability of sgRNA off-target propensity prediction
Source: BMC Bioinformatics. 2020 Feb 10;21:51. doi: 10.1186/s12859-020-3395-z (PMC7011380; doi:10.1186/s12859-020-3395-z)
Supplement: Supplementary file 2 — Additional file 2. A detailed description of the model structure for model selection. [file 12859_2020_3395_MOESM2_ESM.pdf]

A detailed description of the model structure.

Table S1. Structure of **CnnCrispr** prediction model.

**Layer**	**Description**	**Output size**
Embeding	Input size = 16×100	23×100
BiLSTM	Units=40	23×80
Convolution	Filters=10, kernel size=5	19×10
Batch Normalization		
Convolution	Filters=20, kernel size=5	15×20
Batch Normalization		
Convolution	Filters=40, kernel size=5	11×40
Batch Normalization		
Convolution	Filters=80, kernel size=5	7×80
Batch Normalization		
Convolution	Filters=100, kernel size=5	3×100
Batch Normalization		
Flatten		1×300
Dropout		
Dense	Activation=*relu*	1×20
Dropout		
Dense	Activation=*softmax*(Classification)<br>Activation=*sigmoid*(Regression)	1×2<br>1×1

Table S2. Structure of **CnnCrispr_NoLSTM** prediction model.

**Layer**	**Description**	**Output size**
Embeding	Input size = 16×100	23×100
Convolution	Filters=10, kernel size=5	19×10
Batch Normalization		
Convolution	Filters=20, kernel size=5	15×20
Batch Normalization		
Convolution	Filters=40, kernel size=5	11×40
Batch Normalization		
Convolution	Filters=80, kernel size=5	7×80
Batch Normalization		
Convolution	Filters=100, kernel size=5	3×100
Batch Normalization		
Flatten		1×300
Dropout		
Dense	Activation=*relu*	1×20
Dropout		
Dense	Activation=*softmax*(Classification)<br>Activation=*sigmoid*(Regression)	1×2<br>1×1

Table S3. Structure of **CnnCrispr_Conv_LSTM** prediction model.

Layer	Description	Output size
Embeding	Input size = 16×100	23×100
Convolution	Filters=10, kernel size=5	19×10
Batch Normalization		
Convolution	Filters=20, kernel size=5	15×20
Batch Normalization		
Convolution	Filters=40, kernel size=5	11×40
Batch Normalization		
Convolution	Filters=80, kernel size=5	7×80
Batch Normalization		
Convolution	Filters=100, kernel size=5	3×100
Batch Normalization		
BiLSTM	Units=40	3×80
Flatten		1×240
Dropout		
Dense	Activation=*relu*	1×20
Dropout		
Dense	Activation=*softmax*(Classification)<br>Activation=*sigmoid*(Regression)	1×2<br>1×1

Table S4. Structure of **CnnCrispr_NoBatchNor** prediction model.

Layer	Description	Output size
Embeding	Input size = 16×100	23×100
BiLSTM	Units=40	23×80
Convolution	Filters=10, kernel size=5	19×10
Convolution	Filters=20, kernel size=5	15×20
Convolution	Filters=40, kernel size=5	11×40
Convolution	Filters=80, kernel size=5	7×80
Convolution	Filters=100, kernel size=5	3×100
Flatten		1×300
Dropout		
Dense	Activation=*relu*	1×20
Dropout		
Dense	Activation=*softmax*(Classification)<br>Activation=*sigmoid*(Regression)	1×2<br>1×1

Table S5. Structure of **CnnCrispr_NoDropout** prediction mdoel.

Layer	Description	Output size
Embeding	Input size = 16×100	23×100
BiLSTM	Units=40	23×80
Convolution	Filters=10, kernel size=5	19×10
Batch Normalization		

Convolution	Filters=20, kernel size=5	15×20
Batch Normalization		
Convolution	Filters=40, kernel size=5	11×40
Batch Normalization		
Convolution	Filters=80, kernel size=5	7×80
Batch Normalization		
Convolution	Filters=100, kernel size=5	3×100
Batch Normalization		
Flatten		1×300
Dense	Activation=*relu*	1×20
Dense	Activation=*softmax*(Classification)<br>Activation=*sigmoid*(Regression)	1×2<br>1×1
